# Association of AbobotulinumtoxinA and Skinbooster for Periorbital Wrinkles: A Randomized, Controlled, Double-Blind, Split-Face Study

**DOI:** 10.1007/s00266-026-06059-9

**Published:** 2026-07-08

**Authors:** Ana Claudia Laureano Navarro Shishido, Livia Leão Leite Tostes, Bryanne Brissian de Souza Nobre, Jacqueline Rosa Gonçalves, Lucas Alexandre de Sousa Mendes, Ana Claudia Carbone, Alfonso Sánchez-Ayala, Mariana Barbosa Câmara-Souza, Giancarlo De la Torre Canales

**Affiliations:** 1 Department of Dentistry, Ingá University Center, Uningá, Maringá, Paraná Brazil; 2 Department of Dentistry, Sao Leopoldo Mandic University, Campinas, Espirito Santo Brazil; 3 Department of Dentistry, Federal University of Uberlandia, Uberlandia, Minas Gerais Brazil; 4 Department of Dentistry, University of Ponta Grossa, Ponta Grossa, Paraná Brazil; 5 Araras Dental School, Hermínio Ometto Foundation – FHO, Araras, São Paulo Brazil; 6 Egas Moniz Center for Interdisciplinary Research (CiiEM), Egas Moniz School of Health and Science, Caparica, Almada, Portugal; 7 Division of Oral Diagnostics and Rehabilitation, Department of Dental Medicine, Karolinska, Huddinge, Sweden

**Keywords:** Botulinum toxin type a, Hyaluronic acid, Polymers, Canthal lines

## Abstract

**Background:**

Although multimodal aesthetic treatments are used, the combined effects of botulinum toxin A and hyaluronic acid skinbooster for periorbital rejuvenation have not been properly evaluated. This study assessed the efficacy and durability of associating abobotulinumtoxinA and Restylane^®^ Skinbooster Vital for the treatment of canthal lines.

**Methods:**

Twenty-four Brazilian women with dynamic periorbital wrinkles (Merz 5-point scale, levels II-IV) participated in this randomized, double-blinded, split-face trial. After bilateral abobotulinumtoxinA injections, one side of the face was randomized to receive Restylane^®^ Skinbooster Vital, and the contralateral side underwent a simulated injection. Electromyography (EMG) activity, wrinkle severity, skin texture, and satisfaction with treatments were assessed at baseline and at 1, 2, 3, and 6 months. Data were analyzed using two-way repeated measures ANOVA, Bonferroni’s post-hoc tests, and the Mann-Whitney U test.

**Results:**

EMG showed significant reductions over time, with no differences between treatments protocols (*p* > 0.05). For canthal wrinkle severity, the combined protocol (AbobotulinumtoxinA + Restylane® Skinbooster Vital) demonstrated lower scores at 3 months (*p* = 0.0001) and 6 months (*p* = 0.003) compared with AbobotulinumtoxinA alone, as reported by patients. Skin texture progressively improved following Restylane® Skinbooster Vital application, remaining significantly higher at 3 (*p* = 0.0001) and 6 months (*p* = 0.002) when compared with AbobotulinumtoxinA alone. Patient satisfaction increased in both protocols, with superior results at 3 months in the combined protocol group (*p* = 0.009).

**Conclusion:**

The association of abobotulinumtoxinA with Restylane® Skinbooster Vital provides enhanced and lasting improvements in severe canthal wrinkles.

**Level of Evidence I:**

This journal requires that authors assign a level of evidence to each article. For a full description of these Evidence-Based Medicine ratings, please refer to the Table of Contents or the online Instructions to Authors www.springer.com/00266.

## Introduction

The periorbital region is fundamental to facial expressions and aesthetics, serving as a key determinant of how emotions are perceived [1, 2]. Over time, structural and dynamic changes in this area may produce expressions that unintentionally convey tiredness, unhappiness, or anger, thereby distorting emotional communication and enhancing the perception of aging [3].

Periorbital aging is a multifactorial process that affects all facial layers [4]. Alterations such as bone remodeling and resorption of the orbital septum, orbital fat herniation (with fat loss or descent in regions such as the superomedial orbit), and decreased subcutaneous collagen contribute to common clinical features of aging, including eyelid bags, skin laxity, and wrinkles [1–5]. Additionally, the formation of periorbital wrinkles from repetitive contraction of the orbicularis oculi muscle significantly accentuates the aged appearance of this region [1].

Managing periorbital aging remains a clinical challenge due to its complex, multifactorial nature. Botulinum toxin type A (BoNT-A) and hyaluronic acid (HA) fillers are among the most widely used minimally invasive procedures for facial rejuvenation, including the periorbital area. Furthermore, with increases of 26.9 and 56.4% in procedures since 2020, respectively, BoNT-A and HA rank as the most frequently performed non-surgical aesthetic treatments worldwide according to the 2024 International Society of Aesthetic Plastic Surgery (ISAPS) global survey [6]. Literature consistently supports the efficacy of BoNT-A in improving dynamic wrinkles of the periorbital region, regardless of the commercial brand used [7, 8]. However, as wrinkles progress and become static, a combined treatment strategy that integrates fillers and neuromodulators is often indicated to optimize outcomes and enhance patient satisfaction [9–11].

In such cases, HA fillers can provide significant benefits by restoring volume and improving skin quality [12]. Nevertheless, selecting the appropriate HA is crucial for achieving successful results in the periorbital region, given its high mobility and delicate anatomy. HA products with lower degrees of crosslink, commonly referred to as “skinboosters”, such as Juvederm® VYC-12 (currently marketed as Skinvive) and Restylane® Skinbooster “Vital” and “Vital Light”, are preferred for enhancing fine skin texture, as they reinforce and support the extracellular matrix [13, 14]. Findings from a previous study by our group demonstrated that the association of onabotulinumtoxinA and Juvederm^®^ VYC-12 significantly improved both resting and dynamic periorbital wrinkles compared with onabotulinumtoxinA alone. However, because skin quality parameters were not assessed, the underlying mechanism related to HA’s contribution to these improvements could not be adequately explored.

Restylane^®^ Skinbooster Vital (NASHA technology) is designed to progressively enhance skin quality through precise intradermal or subdermal microinjections [15]. Its primary indication is the correction of fine and superficial lines [16], thereby improving skin elasticity, surface texture, and collagen production. It is recognized as an effective option for rejuvenating the face and neck, given its capacity to restore hydration and support structural integrity over time [17–20]. Based on these characteristics and its low degree of crosslinking, associating this HA formulation with neuromodulators acting at different tissue levels may provide a synergistic approach for treating severe periorbital wrinkles. Nevertheless, the combined efficacy and durability of such treatments remain insufficiently investigated in randomized clinical trials. Therefore, the present study aimed to evaluate the efficacy and durability of a combined treatment protocol using abobotulinumtoxinA and Restylane^®^ Skinbooster Vital in individuals with moderate-to-severe periorbital wrinkles.

## Methods

This randomized, double-blind, split-face clinical trial was conducted at a specialized aesthetic clinic in Brazil between March and October 2025 (last follow-up). The study protocol received ethical approval from the Research Ethics Committee of Uninga University (CAAE: 81365624.1.0000.5220) and adhered to the principles outlined in the Declaration of Helsinki. All procedures were conducted in accordance with the CONSORT guidelines. Written and signed informed consent was obtained from all participants prior to enrollment in the study and for the publication of any potentially identifiable images included in this article.

### Participants and Sample Size

This trial enrolled Brazilian female participants aged 40 to 70 years who reported aesthetic dissatisfaction with dynamic periorbital wrinkles. Eligibility required wrinkles severity grades II to IV on the Merz 5-point scale [21]. Exclusion criteria included prior BoNT-A treatments (cosmetic or therapeutic) or other aesthetic interventions (surgical or non-surgical) performed in the upper facial third within the preceding 12 months. Participants with autoimmune or neuromuscular diseases, those who had received vaccines within three months before baseline, or who were using medications known to interfere with neuromuscular transmission were also not eligible.

The sample size was estimated using G*Power 3.1.9.2 software (version 3.1.9.2, Kiel, Germany). A pilot study (*n* = 4 per group) of orbicularis oculi electromyographic activity was used to estimate the effect size, adopting a repeated-measures ANOVA model with within- and between-subjects interactions. The following parameters were applied: *α* = 0.05, power = 0.8, two groups, five measurements, and an estimated effect size (f) of 0.427. The computation yielded a total of 40 face sides (20 participants). To compensate for an anticipated 20% loss rate, a final target of 50 face sides (25 subjects) was established for allocation across study arms.

### Study Protocol

Participants underwent six study visits. During the first visit, eligibility was determined according to the inclusion and exclusion criteria. At the second visit, baseline assessments were conducted, followed by BoNT-A injections. One month later (Visit 3), participants were randomized by facial side: one side received HA injections while the contralateral side underwent a simulated injection (cannula insertion and technique replication without gel delivery or other subtance). Subsequent evaluations were performed at 2, 3, and 6 months after the initial BoNT-A treatment.

### Randomization and Blinding

Participants’ faces were randomly assigned in blocks of two using an online computer-generated sequence (http://www.randomization.com/), managed by an independent technician not otherwise involved in the study. For each participant, the technician prepared a sealed, opaque envelope indicating which side of the face would receive the HA injection. This information remained concealed from both the evaluating researcher (A.C.N) and the participants until data collection was completed. All aesthetic procedures were performed by an experienced clinician specialized in injectables (B.B.N), who was not involved in the assessment of the outcomes, analysis or any other phase of the study. Face sides were randomized as follows: AbobotulinumtoxinA (AboBTX, *n* = 24) and Abobotulinumtoxin A + Restylane® Skinbooster Vital (AboBTX-SkBO, *n* = 24). Blinding effectiveness was assessed at the last follow-up by asking the participants to identify the facial side they believed had received the associated treatment. None of the participants correctly identified the treatment allocation, suggesting successful maintenance of blinding throughout the study.

### Interventions

BoNT-A injections were administered in accordance with the international consensus guidelines for abobotulinumtoxinA [22]. Each vial of BoNT-A (Dysport/abobotulinumtoxinA, 500 U, Ipsen, Wrexham, United Kingdom) was reconstituted with 2 mL of 0.9% isotonic sterile saline solution, yielding a final concentration of 25 s.U/0.1 mL. A total dose of 60 s.U was injected into the orbicularis oculi muscles per participant, distributed across three injection points per side (10 s.U each/30 s.U total). Injections were delivered in a medial-to-lateral direction, anterior to the zygomatic bone prominence, using 31G x 6mm needles. No additional injection points were employed throughout the study.

For HA treatment, Restylane Skinbooster Vital® (NASHA, Galderma, Uppsala, Sweden) was injected in the periorbital region. A total volume of 0.5 mL per site was administered into the superficial subcutaneous plane in micro-bolus, using a retrolinear threading technique aligned with the direction of periorbital lines (three passes were performed). On the contralateral site, a simulated injection was performed by inserting a cannula and reproducing the same technique without delivery of HA or any other product. Injections were performed specifically spanning the area between the lateral brow tail and the zygomatic arch. To preserve masking, participants were unable to discern whether HA was injected. Both HA and simulated procedures were performed using a 22G x 50mm cannula.

### Outcomes

Outcomes were evaluated at five time points: baseline and 1, 2, 3, and 6 months after BoNT-A injections. All objective and subjective assessments were performed by investigators who were unaware of treatment allocation and were not involved in any other aspect of the study. The primary outcome of the study was the electromyographic activity of the orbicularis oculi muscles. Secondary outcomes included the severity of canthal lines, skin texture, and patient-reported satisfaction following treatments.

### Electromyography (EMG)

EMG recordings were obtained using the Quattro+ system (OT Bioelettronica, Turin, Italy), a multichannel amplifier designed for high-fidelity acquisition of bioelectric signals. The system enables bipolar EMG recordings with a built-in inertial measurement unit (IMU) for synchronized motion and muscle activity tracking (sampling rates 2000–3000 Hz, amplification 150×, and band-pass filtering between 20–900 Hz). Bipolar Ag/AgCl surface electrodes were positioned over the orbicularis oculi after cleaning the skin with 70% alcohol, and the reference electrode was placed on the wrist. Muscle activity was recorded simultaneously on both sides during maximum voluntary contraction (MVC), elicited by forceful eye closure combined with smiling to ensure maximal recruitment of the orbicularis oculi. Each contraction was sustained for 5 s and repeated three times with 2 min rest intervals to prevent fatigue. The root-mean-square (RMS) values of the EMG signal were calculated, and the mean RMS value across the three MVC recordings was used for statistical analysis.

### Severity of Canthal Lines

Wrinkle severity was assessed using the validated Merz 5-point Crow’s Feet Grading Scale [21]. This scale rates wrinkle severity in both relaxed and maximal contraction states as follows: 0 = no lines; 1 = mild; 2 = moderate; 3 = severe; 4 = very severe. Patients and evaluators rated the severity of canthal lines by visual inspection during each follow-up visit.

### 3D-Stereophotogrammetry

3D Skin surface analysis was performed using a three-dimensional stereophotogrammetric system (VECTRA^®^ H2; Canfield Scientific, Fairfield, NJ, USA). Participants were seated upright, maintaining a neutral head position (Frankfort horizontal plane parallel to the floor) and a relaxed facial expression. The images were processed with the FACE SCULTOR^®^ software (Canfield Scientific), which generates quantitative maps of the selected skin region surface characteristics. The analysis focused on skin texture uniformity in the periorbital region. All acquisitions were conducted under controlled and standardized conditions.

### Patient’s Perceived Satisfaction with BoNT-A Treatment

Patient satisfaction with BoNT-A treatment was assessed using the *FACE-Q Crow’s Feet Module* [23]. This validated instrument measures the degree of bother caused by periorbital lines during the preceding week, rated on a 4-point Likert scale (1 = not at all; 2 = a little; 3 = moderately; 4 = extremely). The raw scores were summed and converted to a standardized scale from 0 (worst possible outcome) to 100 (best possible outcome) using established conversion tables.

The authors obtained a license agreement to use the FACE-Q scales for non-commercial academic research purposes.

### Statistical Analysis

Data analysis was performed using IBM SPSS Statistics (version 25; IBM Corp., Armonk, NY, USA). All tests were two-tailed, with a significance level of *α* = 0.05 and power (1 – β) = 0.80. Normality and homogeneity of variances were verified using the Shapiro-Wilk and Levene tests, respectively. Sphericity was assessed with Mauchly’s test. Changes in EMG activity, FACE-Q satisfaction, and skin texture scores over time and between treatments were analyzed using two-way repeated-measures ANOVA, with Bonferroni correction applied for post-hoc comparisons. Wrinkle severity scores were compared using the Mann-Whitney U test.

## Results

### Study Population

**Fig. 1:**
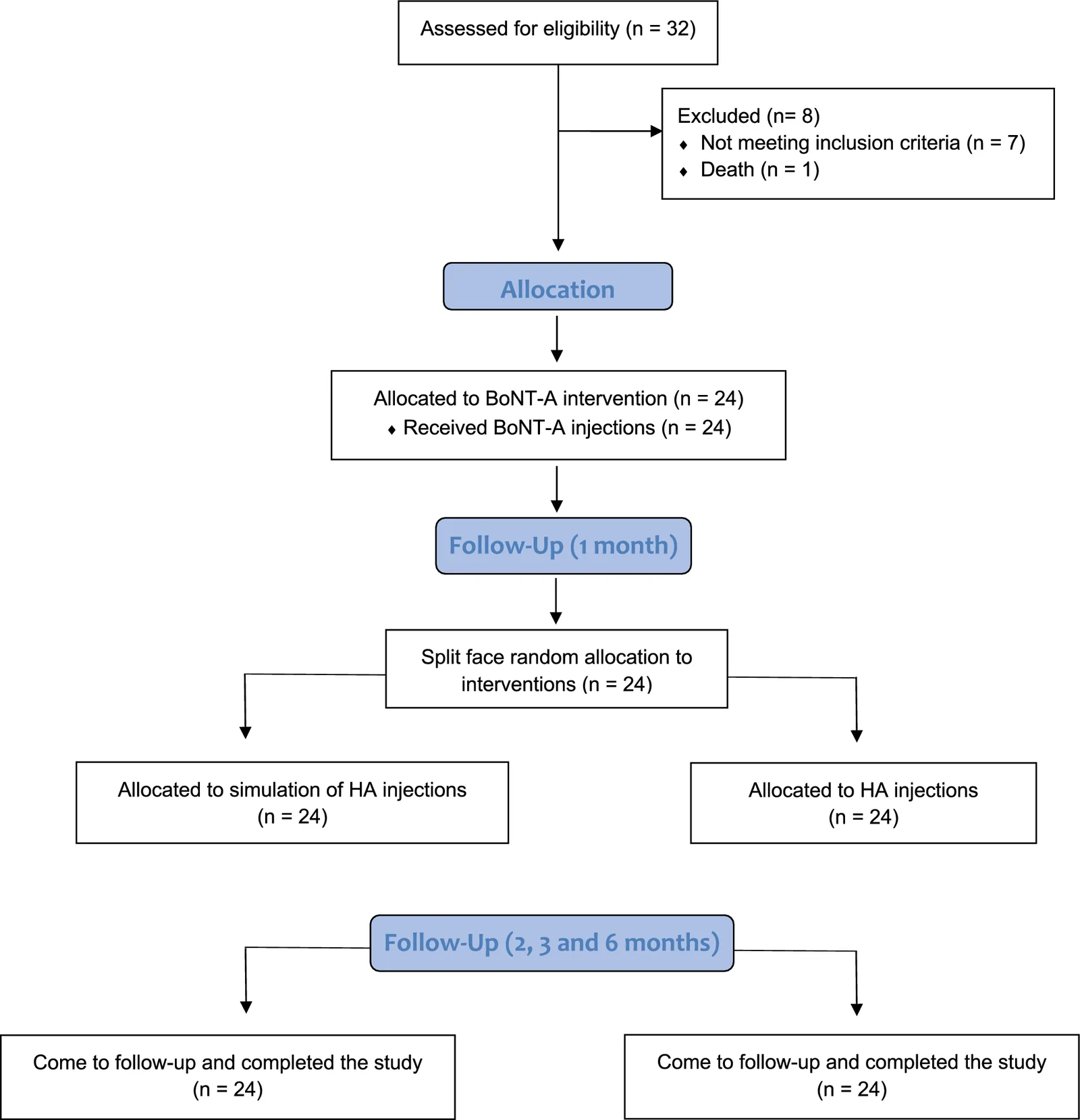
CONSORT diagram

A total of 32 Brazilian women were screened for eligibility. Seven participants did not meet the inclusion criteria and were excluded prior to enrollment. One additional participant was excluded due to death from causes unrelated to the study, before receiving any intervention or participating in follow-up assessments, resulting in a final sample of 24 enrolled patients (46.41 ± 8.31). All participants completed the six-month follow-up period (Fig. 1). At baseline, periorbital wrinkles were distributed as follows: 13 patients (54.2%) presented with very severe lines, 3 (12.5%) with severe lines, and 8 (33.3%) with moderate lines.

### Electromyographic Activity

**Fig. 2:**
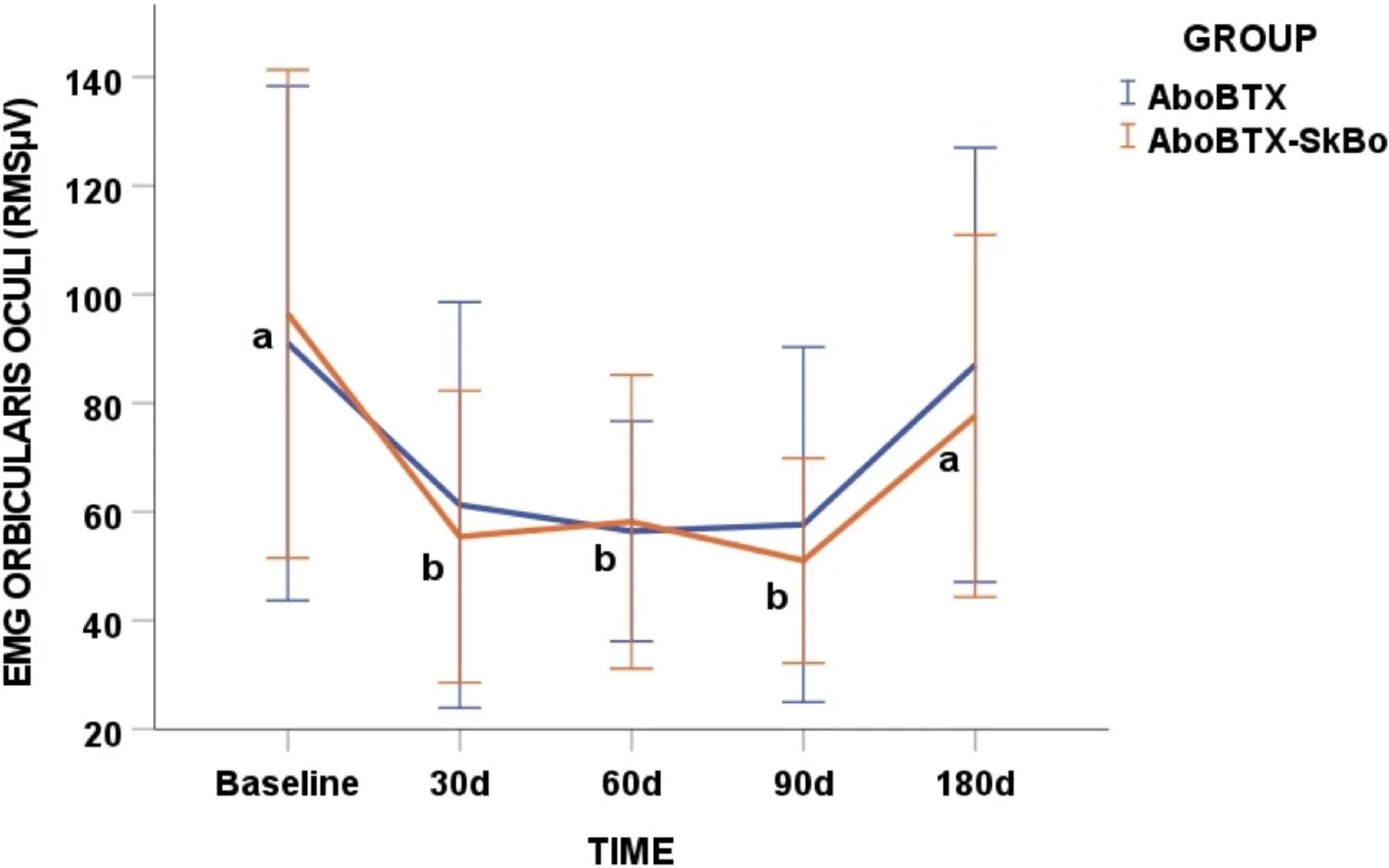
Changes in root mean square scores (RMS µV) in maximum voluntary contraction. (MVC) of orbicularis oculi according to treatments and timepoints. Different lower-case letters mean time points with a significant difference at 0.05. No inter-group differences were found in the study

Both treatment protocols produced significant reductions in the electrical activity of the orbicularis oculi muscle at 1-, 2-, and 3-month evaluations compared with baseline (*p* = 0.002). No significant differences were observed between baseline and the 6-month follow-up (*p* = 1.0). Despite these intra-group improvements, no significant differences emerged between the two protocols at any of the assessed time points (*p* > 0.05), confirming a comparable EMG response across treatments. (Fig. 2).

### Canthal Lines Severity

**Fig. 3:**
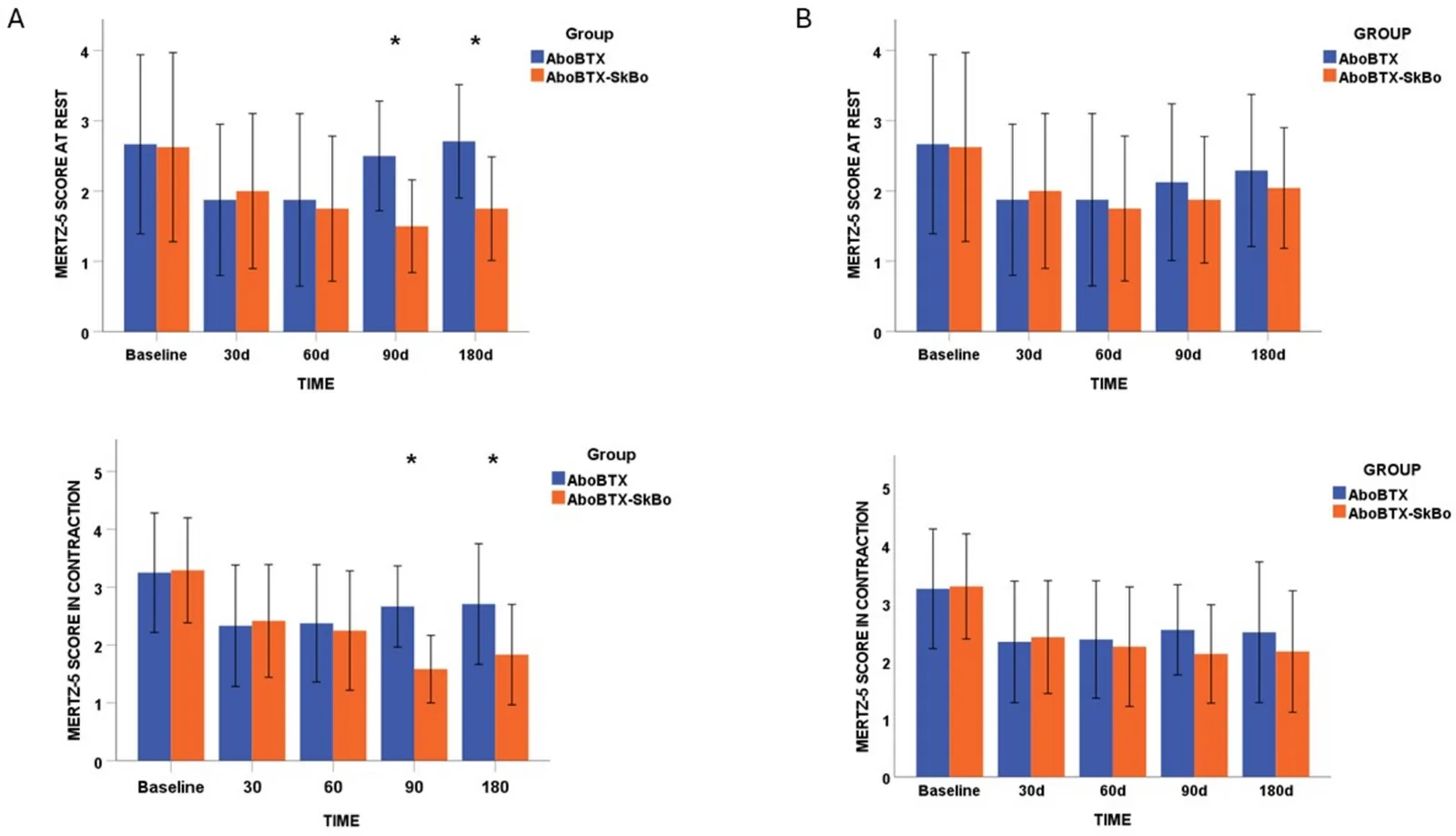
Severity of canthal lines (Merz scale scores) in rest and contracted positions according to treatment and time points. **A** patients’ assessment; **B** evaluators’ assessment. *Inter-group significant differences (*p *< 0.05)

**Fig. 4:**
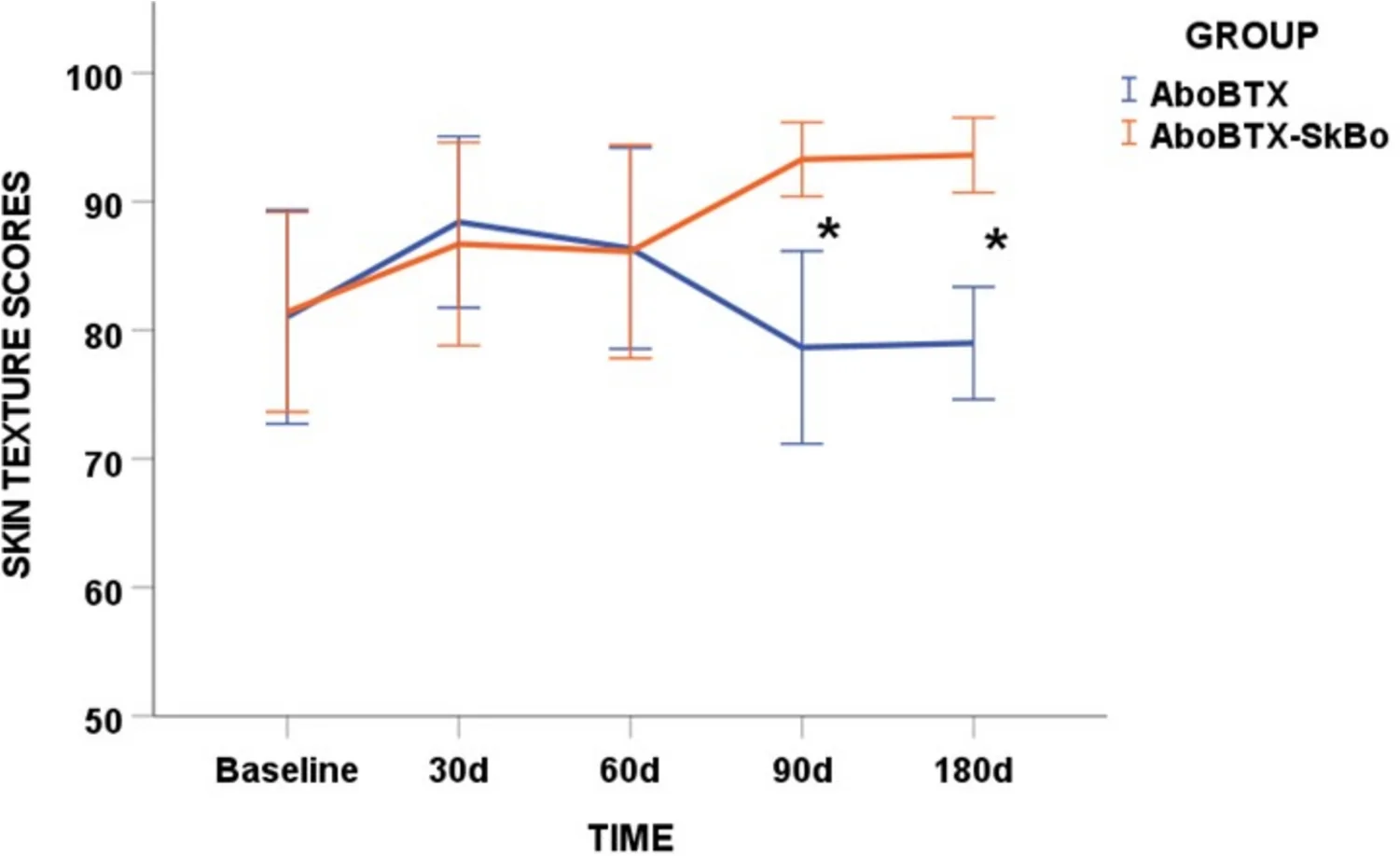
Skin texture scores by treatment and time points. *Inter-group significant differences (*p *< 0.05)

Patient-reported canthal wrinkles severity showed significant reductions following the BoNT-A injection, with improvements maintained up to the 2-month follow-up (*p* = 0.001) at rest and up to the 3-month assessment (*p* = 0.025) at contraction (Fig. 4A). When comparing treatments, the AboBTX-SkBo protocol resulted in significantly reduced wrinkle severity in both rest and contracted positions at the 3-month (*p* = 0.0001) and 6-month (*p* = 0.003) assessments (Fig. 3A).

Evaluator-reported periorbital wrinkles severity also showed a consistent significant reduction at rest and during contraction for both protocols until the 3-months assessment compared with baseline (*p* < 0.05). However, no significant differences were observed at any time point between protocols (*p* > 0.05) (Fig. 3B).

### Skin Texture

**Fig. 5:**
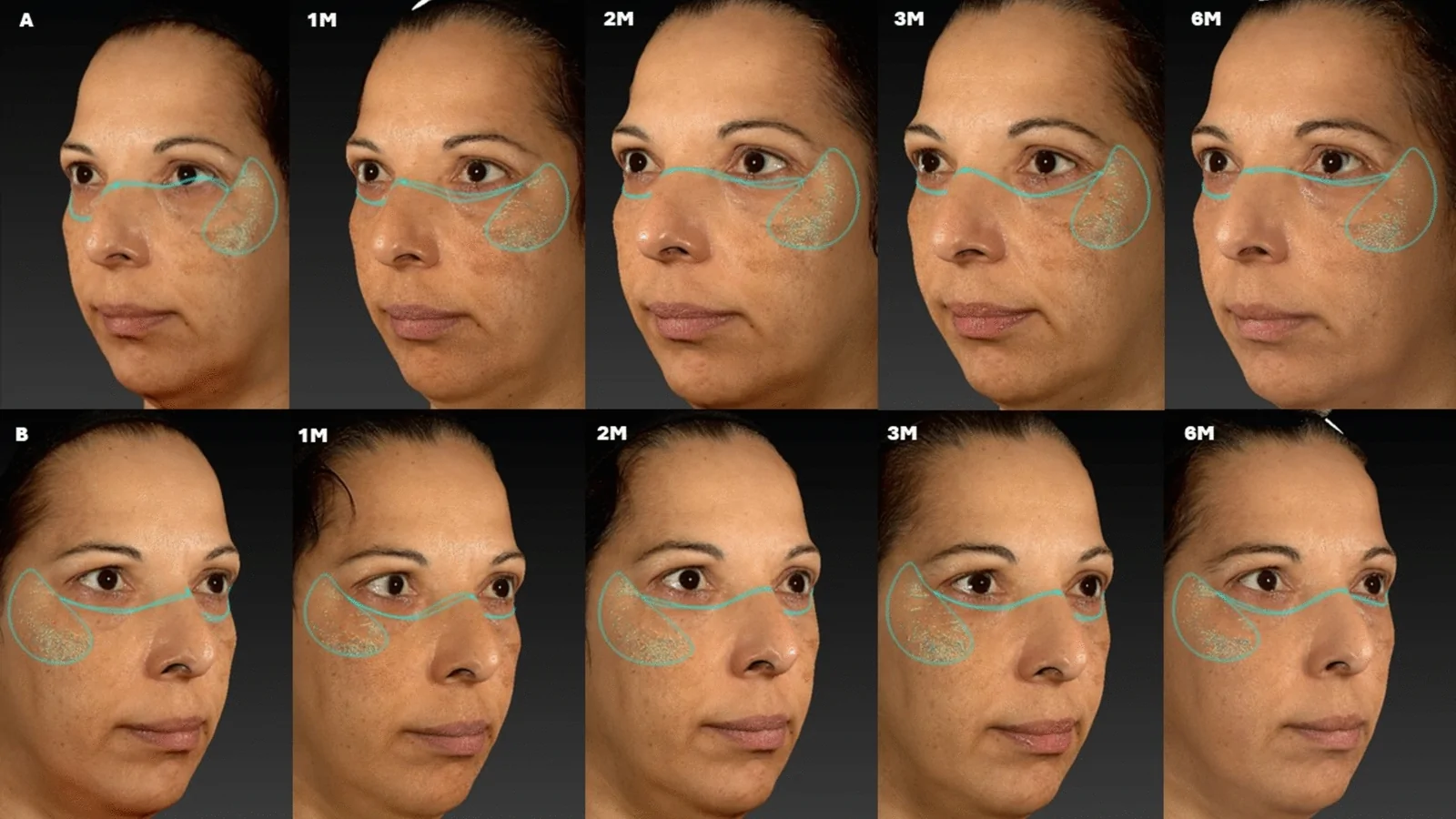
Diagram of the mask used for skin texture assessment of the periorbital region using stereophotogrammetry according to different time points. **A** Side treated with AbobotulinumtoxinA + with Restylane® Skinbooster Vital; **B** Side treated with AbobotulinumtoxinA

The injection of BoNT-A produced significant improvements in skin texture at the 1-month (*p* = 0.002) and 2-month (*p* = 0.04) follow-up visits compared with baseline. However, these gains were not sustained over time in the AboBTX group. In contrast, the AboBTX-SkBo protocol maintained a consistent overall increase in skin texture throughout all follow-up evaluations relative to baseline (*p* = 0.001). When comparing groups, the AboBTX-SkBo protocol demonstrated significantly higher skin texture scores at 3 (*p* = 0.0001)- and 6-month (*p* = 0.002) assessments (Fig. 4). Figure 5 shows the skin texture results of one case treated in this study.

### Patient Satisfaction with Treatment

**Fig. 6:**
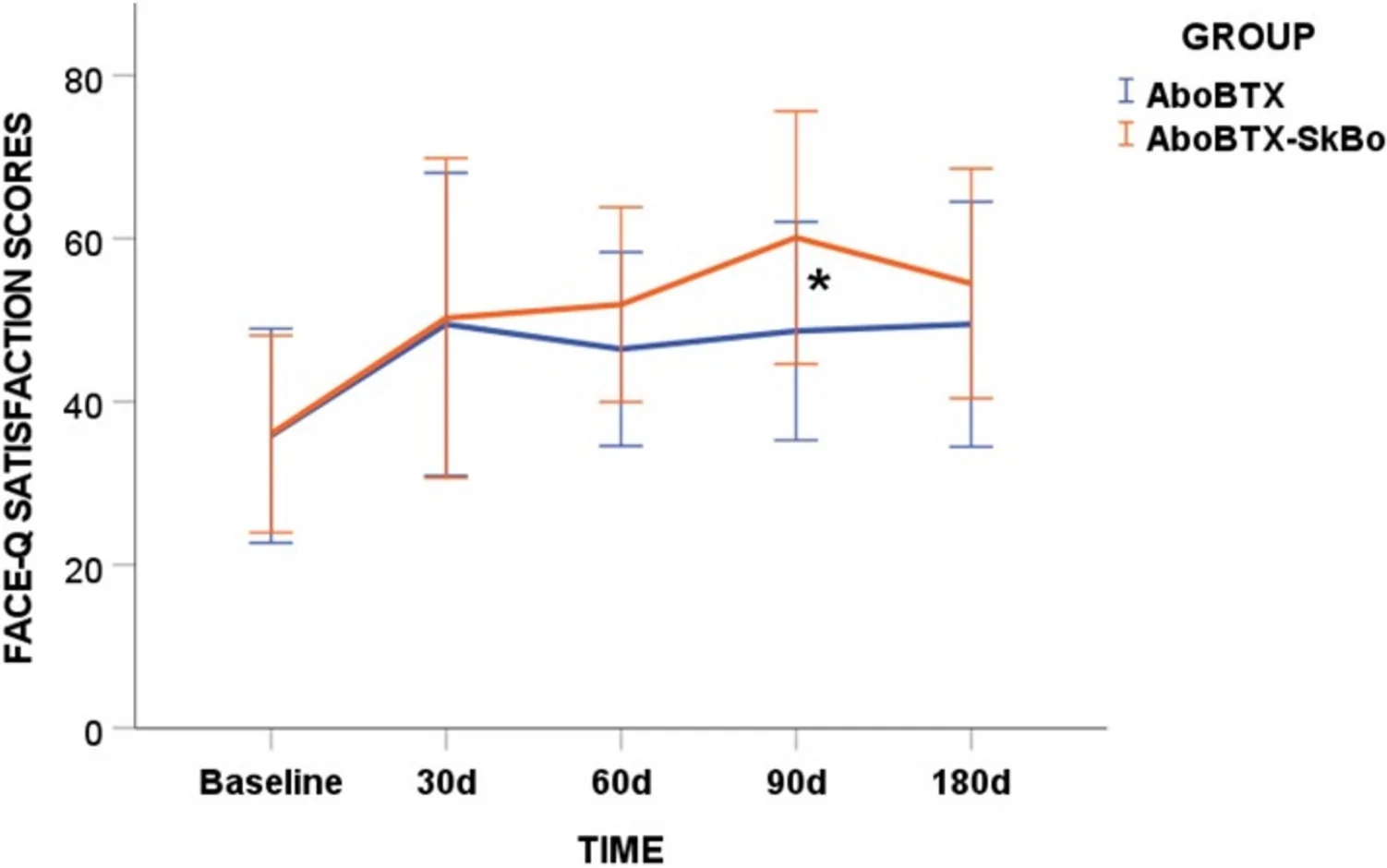
Face-Q satisfaction with the treatment scores by treatment and time points. *Inter-group significant differences (*p *< 0.05)

Both treatment protocols led to significant increases in patients’ satisfaction with treatment at the 2-month follow-up compared with baseline (*p* = 0.02). However, only the AboBTX-SkBo protocol demonstrated additional significant improvements at the 3-month (*p* = 0.001) and 6-month (*p* = 0.001) assessments compared to baseline. When comparing the protocols, only the 3-month assessment showed a difference: satisfaction was significantly higher in the AboBTX-SkBo treatment (*p* = 0.009) than in AboBTX-only (Fig. 6).

### Adverse Events

Mild and expected procedure-related reactions were observed following Restylane^®^ Skinbooster Vital injections, predominantly transient pain in 29.1% participants and small hematomas in 12.5% of the participants at the injection sites. All events resolved spontaneously within two weeks without the need for additional intervention, and no serious adverse events were reported in either treatment group.

## Discussion

Given the aesthetic sensitivity, the functional relevance of the periorbital area, and its early susceptibility to aging changes, establishing clinically validated protocols to enhance periorbital rejuvenation with predictable, durable results remains a central priority in clinical aesthetic practice. In this context, the present study provides novel clinical data by evaluating the combined use of abobotulinumtoxinA and Restylane^®^ Skinbooster Vital for the management of severe canthal wrinkles. This combined strategy, produced more consistent and longer-lasting improvements in wrinkle severity, patient satisfaction, and skin texture than abobotulinumtoxinA alone. Importantly, the treatment was well tolerated, and no severe adverse effects were observed throughout the study period, which is in line with the solid safety profile of Restylane® fillers [24].

Our results indicate that the reduction in periorbital wrinkle severity observed after abobotulinumtoxinA injections is closely associated with the decrease in orbicularis oculi muscle activity, which remained evident through the 3-month evaluation. This finding is in line with previous studies reporting a diminution of abobotulinumtoxinA effects after 3-4 months [25]. This attenuation in muscular contraction was mirrored by patients’ and evaluators’ subjective perception of improvement, as reflected in the Merz 5-point crow’s feet scale data. Comparable patterns have been consistently reported in studies employing abobotulinumtoxinA [26–28]. Moreover, our data aligned with broader literature indicating that the aesthetic effect of BoNT-A on canthal wrinkles tends to be similar across different brands, a point demonstrated in investigations involving incobotulinumtoxinA, onabotulinumtoxinA, and relabotulinumtoxinA [25, 29–32]. Unlike previous studies, incorporating EMG into our research supports subjective assessments and enhances the reliability of the findings.

Furthermore, patient-reported improvements in periorbital wrinkle severity persisted at the six-month follow-up in the AboBTX-SkBo protocol, even though EMG activity of the treated muscle had returned to baseline levels. This dissociation between muscular activity and perceived wrinkle reduction suggests that the sustained clinical benefit observed in the combined-treatment group may be driven less by residual neuromodulatory action and more by the cutaneous enhancements promoted by Restylane^®^ Skinbooster Vital. As a stabilized HA microinjection system designed to improve hydration, elasticity, and overall dermal quality, Restylane^®^ Skinbooster Vital has been shown to enhance superficial lines and skin texture in facial areas (periorbital, perioral, and cheeks) by increasing skin firmness and improving the skin’s elastic recovery capacities [17–19]. The skin texture results of the AboBTX-SkBo, which were more significant since the 3-month follow-up and remained improved at the 6-month follow-up, reinforced these mechanisms and suggested that the modulation of the dermal matrix exerted by Restylane® Skinbooster Vital reduces the recurrence rate of severe wrinkles. In addition, it has been shown that NASHA fillers increased production of Type 1 procollagen and upregulate gene expression for Types I and II procollagen [17]. These dermal changes likely reduce the visual prominence of periorbital wrinkles, even when neuromodulatory action is no longer prominent, thereby explaining the prolonged patient-perceived improvement noted in our study. Although evaluators’ reports did not reveal significant differences between protocols at the 3- and 6-month time points, the AboBTX-SkBo protocol still demonstrated higher scores in periorbital wrinkle severity at these time points. Comparing the patients’ and evaluators’ findings, these differences may suggest that patients are more sensitive to subtle aesthetic changes or that their perception of changes with the treatment last longer since they reported improvements for six months. In contrast, evaluators may apply more conservative or demanding criteria when assessing aesthetic outcomes, which could explain why improvements reported after 6 months were not statistically significant compared with baseline.

The aforementioned results are consistent with the findings of our previous investigation using an association of onabotulinumtoxinA and VYC-12 (Vycross technology), indicated to improve skin hydration and elasticity, in which we similarly observed prolonged positive effects on wrinkle severity when both treatments were used in tandem [31]. Together, these data support the notion that pairing a neuromodulator with a HA produces longer-lasting aesthetic benefits than the neuromodulator alone, underscoring the clinical relevance of integrating both treatments for periorbital rejuvenation. Since both VYC-12 and Restylane® Skinbooster Vital rely on distinct HA technologies, the question of which formulation provides the greatest and most durable enhancement when associated with a neuromodulator remains open.

Patient satisfaction is a key indicator of treatment success, influencing both clinical outcomes and long-term engagement [33]. Ensuring that the most appropriate protocol is selected during the first appointment can enhance patients’ perceptions of their results, a concept supported by evidence showing that multimodal aesthetic approaches consistently yield higher levels of satisfaction [10, 11]. This rationale helps explain the superior satisfaction observed in the AboBTX-SkBo group, where the complementary actions of two treatments -one targeting muscle activity and the other enhancing dermal quality- likely produced a more harmonious and natural periorbital appearance. Importantly, none of the patients experienced complete elimination of their wrinkles, a feature that aligns with current aesthetic trends for subtle refinement rather than complete effacement of expression lines. Maintaining soft periorbital expressions is perceived as both natural and emotionally congruent [34, 35], which may have contributed to the heightened satisfaction found in our study.

Although the study offers important insights, the exclusive inclusion of female participants limits the generalizability of the findings, as male participants may require different treatment dosages or exhibit distinct periorbital characteristics. Despite this limitation, the study presents notable strengths. Its robust methodological design enhances the reliability of the outcomes. Also, integrating subjective and objective assessments, including recommendations from a previous study on skin quality [31], provides a multidimensional assessment. Moreover, although the association of abobotulinumtoxinA and Restylane® Skinbooster Vital is performed by clinicians, there were no scientific studies assessing the effects of this combination for periorbital rejuvenation, which add valuable clinical evidence to the field. In addition, the present study indicates that associating abobotulinumtoxinA and Restylane® Skinbooster Vital may reduce the number of HA sessions typically required when the product is used alone, as suggested by previous studies [18]. Although a consensus on Restylane® Skinboosters recommended that the protocol may vary by age and skin quality, our study showed that in middle-aged adults, a single injection session could be sufficient to achieve results up to 3 months [15]. Additionally, since our study classified patients according to wrinkles severity, age cannot be considered a confounder. Taken together, these strengths underscore the relevance of multimodal minimally invasive treatments for patients with severe periorbital wrinkles.

## Conclusion

Associating abobotulinumtoxinA with Restylane® Skinbooster Vital provides a safe, effective, and longer-lasting option for improving severe periorbital wrinkles.

## Data Availability

All data are available upon reasonable request to the corresponding author.
